# Pharmacogenetics Clinical Decision Support Systems for Primary Care in England: Co-Design Study

**DOI:** 10.2196/49230

**Published:** 2024-07-23

**Authors:** Videha Sharma, John McDermott, Jessica Keen, Simon Foster, Pauline Whelan, William Newman

**Affiliations:** 1 Centre for Health Informatics Division of Informatics, Imaging and Data Science University of Manchester Manchester United Kingdom; 2 Pankhurst Institute for Health Technology Research and Innovation University of Manchester Manchester United Kingdom; 3 Manchester Centre for Genomic Medicine St Mary’s Hospital Manchester University NHS Foundation Trust Manchester United Kingdom; 4 Division of Evolution, Infection and Genomics School of Biological Sciences University of Manchester Manchester United Kingdom

**Keywords:** personalized medicine, genomic medicine, pharmacogenetics, user-centred design, medical informatics, clinical decision support systems, side effect, information technology, data, primary care, health informatic

## Abstract

**Background:**

Pharmacogenetics can impact patient care and outcomes through personalizing the selection of medicines, resulting in improved efficacy and a reduction in harmful side effects. Despite the existence of compelling clinical evidence and international guidelines highlighting the benefits of pharmacogenetics in clinical practice, implementation within the National Health Service in the United Kingdom is limited. An important barrier to overcome is the development of IT solutions that support the integration of pharmacogenetic data into health care systems. This necessitates a better understanding of the role of electronic health records (EHRs) and the design of clinical decision support systems that are acceptable to clinicians, particularly those in primary care.

**Objective:**

Explore the needs and requirements of a pharmacogenetic service from the perspective of primary care clinicians with a view to co-design a prototype solution.

**Methods:**

We used ethnographic and think-aloud observations, user research workshops, and prototyping. The participants for this study included general practitioners and pharmacists. In total, we undertook 5 sessions of ethnographic observation to understand current practices and workflows. This was followed by 3 user research workshops, each with its own topic guide starting with personas and early ideation, through to exploring the potential of clinical decision support systems and prototype design. We subsequently analyzed workshop data using affinity diagramming and refined the key requirements for the solution collaboratively as a multidisciplinary project team.

**Results:**

User research results identified that pharmacogenetic data must be incorporated within existing EHRs rather than through a stand-alone portal. The information presented through clinical decision support systems must be clear, accessible, and user-friendly as the service will be used by a range of end users. Critically, the information should be displayed within the prescribing workflow, rather than discrete results stored statically in the EHR. Finally, the prescribing recommendations should be authoritative to provide confidence in the validity of the results. Based on these findings we co-designed an interactive prototype, demonstrating pharmacogenetic clinical decision support integrated within the prescribing workflow of an EHR.

**Conclusions:**

This study marks a significant step forward in the design of systems that support pharmacogenetic-guided prescribing in primary care settings. Clinical decision support systems have the potential to enhance the personalization of medicines, provided they are effectively implemented within EHRs and present pharmacogenetic data in a user-friendly, actionable, and standardized format. Achieving this requires the development of a decoupled, standards-based architecture that allows for the separation of data from application, facilitating integration across various EHRs through the use of application programming interfaces (APIs). More globally, this study demonstrates the role of health informatics and user-centered design in realizing the potential of personalized medicine at scale and ensuring that the benefits of genomic innovation reach patients and populations effectively.

## Introduction

The effectiveness, safety, and tolerability of medicines vary considerably across the population, a phenomenon that can be challenging for health care systems, clinicians, and patients. There are several factors that contribute to this including the chosen dosing strategy, the accuracy of the initial diagnosis, or individual factors such as medical comorbidities, age, polypharmacy, and adherence issues [1]. There is now strong evidence that common genetic variation also plays a significant role, a concept known as pharmacogenetics [2].

There are many laboratory technologies that can be used for pharmacogenetic testing. The challenge is returning these results to physicians in a clinically relevant format and clinically relevant timeframe at the point of prescribing. Integrating the results of a pharmacogenetic test within a model of service delivery to inform prescribing practice (hereafter “pharmacogenetics”) could lead to more accurate prescribing, improving outcomes for patients and ensuring better use of health care resources [3]. At many centers globally, pharmacogenetics is part of routine clinical practice. Although there is notable heterogeneity in the design and implementation of these programs, most make use of pharmacogenetic gene panels as their modality for testing [4]. These panels can survey a range of genes simultaneously, meaning the data produced has utility beyond a single prescription at a single time point.

**Figure 1:**
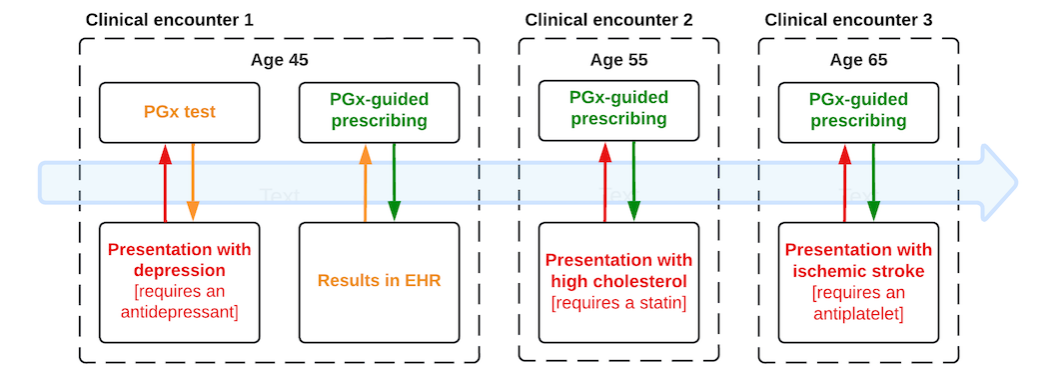
Pharmacogenetic data collected through panel testing may be used throughout the lifetime of a patient at repeated clinical encounters. PGx: pharmacogenetics.

Consider a 45-year-old patient presenting to their primary care physician requiring an antidepressant medicine in March 2023. Providing the genetic data can be returned to the clinician in a readily interpretable format and in a relevant timeframe, the patient’s therapy could be optimized [5]. However, the patient might go on to present in 2033 at 55 years of age, requiring statin therapy for high cholesterol. That pharmacogenetic test, undertaken a decade earlier, could be used to guide the selection and dosing of the patient’s statin [6]. As such, for pharmacogenetics to deliver maximal benefit, the data must be stored and readily accessible throughout a patient’s life (Figure 1).

Many institutions have approached the challenge of persisting data across an individual’s life by integrating pharmacogenetic data within their electronic health care records (EHRs) [7]. This approach makes use of EHR clinical decision support functionality, including alerts or pop-ups. These trigger when a medicine is prescribed where there are clinically actionable pharmacogenetic prescribing recommendations available in that patient’s records. We recently published an international review of pharmacogenetics implementation programs and identified that most are undertaken at specialist centers, primarily based in the United States where health care institutions typically use a single EHR provider [4]. This consistency removes certain challenges related to the interoperability of any solution, something which will be a key issue in the National Health Service (NHS, United Kingdom) where there is a diverse EHR landscape with multiple vendors [8].

At the time of writing, there has been only limited implementation of panel-based pharmacogenetic testing in the NHS. The underlying reasons behind this have been discussed extensively in the past, but a lack of awareness of pharmacogenetics and an inability to return data to clinicians in a suitable format are 2 key barriers [9]. The majority of prescriptions are issued in primary care, and previous studies have suggested that clinicians in this setting are enthusiastic to embrace pharmacogenetic-guided prescribing [10,11]. Although there has been some research into the potential of pharmacogenetics in primary care, there is no granular understanding of how primary care prescribers might want to interact with these data in their EHRs [12]. As an increasing volume of pharmacogenetic data is generated across the next decade, understanding stakeholder preferences, and designing clinical decision support systems for the presentation of these data will be essential to ensure uptake of pharmacogenetic-guided prescribing is maximized.

This study aims to apply a co-design approach to understand how pharmacogenetics might be best implemented in clinical practice in primary care. The objectives of this study were to (1) understand the requirements of a pharmacogenetic service from the perspective of primary care clinicians and (2) use these functional requirements to iteratively design interactive prototypes. The overall aim was to use these findings and prototypes to inform the design and technical architecture of a scalable service that can deliver pharmacogenetics across a range of diverse health care organizations in the NHS.

## Methods

### Overview

This was an iterative co-design study that gathered qualitative data to inform the user interface and user experience for prescribers using pharmacogenetics in primary care. Co-design of digital health interventions supports the active participation of the target end users throughout the development of the intervention aiming to ensure that the end product matches user needs. Co-design has been described as supporting “collective creativity” and is increasingly advocated across the digital health technologies landscape [13,14]. We used ethnographic observations using the “think aloud” method to capture current practices. We subsequently ran workshops with primary care clinicians to explore how pharmacogenetics may be introduced into the workflow and iteratively prototyped a solution that prioritized safety and effectiveness. The overall aim was to better understand perceived barriers to the real-world implementation of pharmacogenetics and identify strategies that would promote adoption.

### Context

This study was the NHS North West Genomic Medicine Service Alliance based in Manchester, United Kingdom. The alliance hosts 1 of the 7 Genomic Laboratory Hubs and serves a wide geographical area covering a population of over 9 million citizens. This study was part of a wider project titled “Pharmacogenetics Roll Out: Gauging Response to Service” (PROGRESS), which is a pilot program to implement pharmacogenetic testing at 4 preliminary primary care centers in the North West England.

### Participants

The target end users for delivering pharmacogenetics were expected to be general practitioners (GPs) and primary care pharmacists. We therefore advertised for participants within these communities through national interest groups and social media platforms. In addition to open advertising, we used mailing lists of clinical networks in primary care and pharmacies covering the whole of the United Kingdom. We received a total of 88 email responses from interested participants. Inclusion and exclusion criteria for selecting participants are summarized in Textbox 1.

For think-aloud ethnographic observations, we selected participants with whom the research team had a previous research relationship. This ensured we had active initial engagement and an even representation of professional and personal backgrounds and participation from end users across a wide geography within the United Kingdom. For workshops, we emailed the pool of interested participants with the date and time of the workshops. We were keen to include participants with little previous experience and or expertise in pharmacogenetics. We included all eligible participants that expressed interest and workshop attendance was determined by who could attend the dates or times set.

**Participant inclusion and exclusion criteria for the co-design process.**

**Inclusion criteria**

- Clinical area
  Primary care and community pharmacy
- Professional role
  General practitioners and pharmacists
- Career stage
  Qualified general practitioners, fully registered pharmacists, and general practitioner specialty trainees
- Geography
  United Kingdom-wide

**Exclusion criteria**

- Clinical area
  Hospital care and social care
- Professional role
  Other health care professionals and patients
- Career stage
  Foundation doctors on general practitioner rotation and preregistration pharmacists
- Geography
  International

### Co-Design

A co-design approach was adopted by the multidisciplinary project team to (1) understand the system end users by identifying their needs, goals, and the context of prescribing in primary care, (2) identify end-user requirements for using pharmacogenetics-based prescribing in their daily practice, (3) understand how pharmacogenetics can best be integrated into the clinical workflow, and (4) identify the barriers to embedding pharmacogenetics-based prescribing into routine practice.

Co-design allows expertise from multiple stakeholders to organically contribute to understanding a problem and developing novel solutions. Co-design aims to move from designing for users to designing with users. It relies on participatory creativity, lived experience, and open ideation throughout the design process [15]. With the increasing complexity of health care and the competing interests of stakeholders, there is a growing recognition of how user-centered design methods may be a powerful driver of change [16].

### Ethnographic Observations

We arranged a Zoom (Zoom Video Communications) interview with selected participants to observe their on-screen prescribing workflows and asked them to verbalize their actions and thoughts (“think aloud”). During the interviews, the participants shared their screens, which we recorded for research purposes. We asked the participants to use the sandbox environment of the EHR and observed them logging in, selecting a patient, and prescribing medications, among other routine clinical tasks. We asked them to share any pain points or positive experiences.

Based on the observed workflows, we created an early prototype using Adobe XD, which represented the current EHRs in use in primary care in the NHS. We used the prototype as a starting point in the subsequent workshops and allowed participants to co-design how pharmacogenetics may be best integrated into the user interface and workflows.

### User Workshops

We undertook 3 user workshops using Zoom. Each workshop had a different topic guide and built upon data captured in the preceding one. We purposefully invited new participants to each workshop to allow a range of perspectives to inform our final design. A bespoke slide deck was made for each workshop to facilitate the discussion and following consent from all participants, the meeting was recorded. The titles for the workshops were as follows: (1) workshop 1: personas and brainstorming; (2) workshop 2: workflows and potential for clinical decision support (alerts); and (3) workshop 3: feedback on prototype including alert timing and content.

### Iterative Prototyping

**Figure 2:**
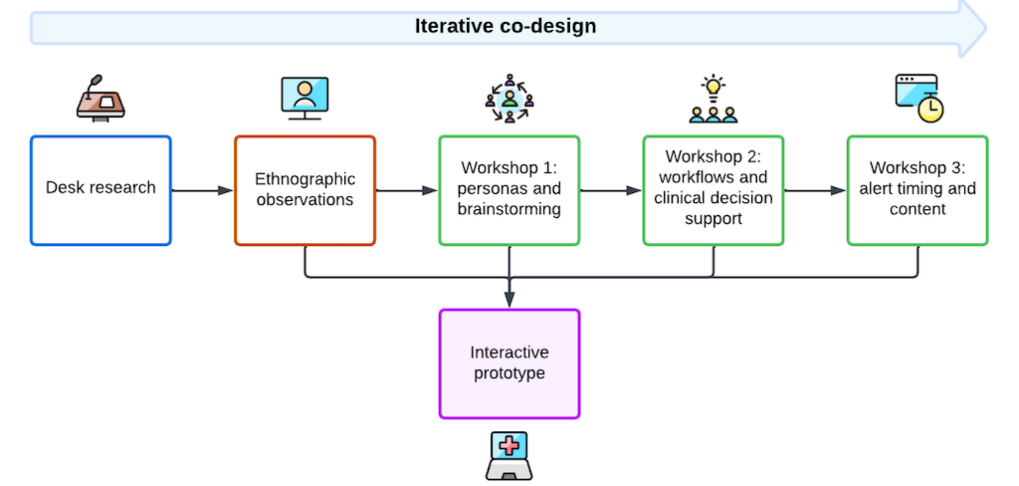
Iterative co-design process demonstrating how different data capture methods were used and co-ordinated to inform the design of an interactive prototype.

Discussion in the workshops was informed by iterative prototyping developed using Adobe XD and presented to participants using the Marvel software system. High-fidelity design prototypes were developed by the team user experience designer based on the ethnographic observations and iteratively refined based on participant feedback after each workshop. The prototypes visualized how a pharmacogenetics-based clinical decision support system could be integrated into an EHR. The prototypes visualized the planned changes to the EHR enabling participants to see how pharmacogenetics components could be displayed and actioned. Figure 2 is a flowchart summary of the iterative co-design process used in this study.

### Agile Data Analysis

The team combined elements of affinity diagramming with a collaborative Agile approach to analyze the detailed notes taken during the workshops. Affinity diagramming is a user research method commonly used to group patterns and themes in qualitative data [17]. The user research team used affinity diagramming to group key themes from the notes after each workshop. Key themes were recorded in Microsoft Excel. After key themes had been identified from the workshop notes, the video recordings were used to revisit the workshops and expand the categories. Key themes and data summaries from the workshop were developed and circulated to the wider research team for review. The project team then met to review and discuss the key themes and workshop insights collectively. Key findings were updated during the group team meeting and were used to inform the preparation of the subsequent workshop (for workshops 1 and 2) and to develop the project report.

### Ethical Considerations

This study voluntarily invited health care professionals to share experiences and perspectives directly related to their routine work. Such research does not require explicit ethical approval as per the NHS Research Ethics Committee decision tool (results of the system attached as Multimedia Appendix 1). All participants provided informed verbal consent before interviews or workshops. Secondary analysis of research data beyond the current study was not included in the consent. All data collected from participants was treated confidentially and stored on the secure research network at the University of Manchester. In addition, all data were deidentified apart from the professional role of participants in relation to the data contributed to this study. All participants were offered compensation for their contribution to the study in the form of a US $40 web-based shopping voucher. Payment rates were set to align with the National Institute for Health and Social Care Research guidance on reimbursement for patients and public contributors to research [18].

### Sample Sizes and Theme Saturation

Research in usability and user research has recommended iterative testing with 5 users per testing round, noting that 85% of usability issues can be identified by 5 users [19]. Although our qualitatively informed approaches in this study did not require definitive sample sizes and we did not design our recruitment strategy to achieve a predefined number of participants, we used the overarching usability samples as guiding principles (5 participants in the ethnographic observations; at least 5 in the workshops). Our overarching aim was to achieve a diversity of engaged practitioners (GPs and pharmacists) at different career stages who could guide us on the design and implementation practicalities of our proposed designs. To ensure our data collection was sufficient to capture the diversity of experience, we did not define the number of user research workshops up front, but instead planned each workshop following preliminary analysis of the previous stage. This enabled the research team to review and discuss findings at each stage and to identify the unknowns that needed further discussion with practitioners in subsequent workshops. The team identified saturation as the point at which no new actionable themes or insights were emerging from the workshops or interviews. When this point was reached, no further data collection activities were conducted.

## Results

### Participants

At the start of each workshop, participants were asked to introduce themselves and provide some background about their experience with pharmacogenetics and digital health. Participants reported varied career stages (eg, from GP trainee to GP with >30 years of experience) and different levels of familiarity with pharmacogenetics and digital health technologies (Table 1). For each co-design element, we included the following participants (total N=24): ethnographic observations – GPs (3) and pharmacists (2); workshop 1 – GPs (3) and pharmacists (2); workshop 2 – GPs (2) and pharmacists (3); workshop 3 – GPs (5) and pharmacists (4).

Four participants used the EMIS Web HER and 1 participant used the SystmOne EHR. Through thinking aloud, users described their experience of logging in and selecting a patient as part of a clinical encounter and described initial frustrations with connectivity and start-up times. One user proactively configured their EHR system with shortcuts, templates, and filters to enhance their workflow. With regards to the prescribing workflow, all users demonstrated a similar on-screen workflow of a pop-up box appearing at the center of the EHR when clicking the “add drug” button. However, users did highlight that the preceding screen from which the prescribing workflow is launched may differ. For example, a user may launch the prescribing workflow from the “current medication” or the “current encounter” screen. From where the user decides to enter the prescribing workflow did not impact the pop-up box or the subsequent prescribing user experience.

We used the recordings of these observational sessions to mock up an EHR for exploring how pharmacogenetics may be introduced into the workflow. This was presented to subsequent workshop participants.

**Table 1:** Summary of participants by career level, pharmacogenetics, and digital health experience.

Workshop	Career level: junior	Career level: mid	Career level: senior	PGx^a^ experience	Digital health experience
1	2	1	2	1	0
2	1	1	3	3	3
3	0	1	8	4	1

^a^PGx: pharmacogenetics.

### Co-Design Workshop Results

A total of 19 people participated across the 3 co-design workshops. Each group co-design workshop lasted 1.5 hours and was facilitated by the lead author of this paper. They were further supported by a clinical geneticist and the user research team (1 senior user experience designer and 1 senior digital health researcher).

The key themes from the 3 workshops are summarized in Table 2. The summary column illustrates how each theme was discussed in the workshop at a high level. The summary is not a direct quote from participants; it is a simplified, anonymized summary of participants’ comments on the theme.

Participants in workshop 1 emphasized the importance of developing a pharmacogenetics-prescribing system that was easily usable by all key prescribers, including health care professionals in training. Integrating the system into the EHR was identified as a mandatory requirement. However, the complexity of clinical prescribing pathways was discussed at length in the workshop. Participants described how prescribing can happen in many ways and that the pharmacogenetics-prescribing system needed to be embedded at multiple points in the EHR to ensure that the recommendations were not missed. The importance and potential of pharmacogenetics to improve patient care was widely recognized by participants. National policy-level incentivization was discussed to ensure that the system would be used, particularly given the busy context of primary care prescribing.

Workshop 2 participants echoed the need expressed in workshop 1 to embed the pharmacogenetics system within the EHR at multiple points, both as alert pop-ups and by integrating pharmacogenetics recommendations into routine prescription reviews. Participants described how prescribers can have different prescribing workflows and how this creates complexity in the implementation of any singular pharmacogenetics-based prescribing approach. Like the comments in workshop 1, clear incentivization to use a pharmacogenetics-based prescribing system was identified as necessary for prescribing teams who are already very busy. Participants identified that the prescribing recommendations should provide direct links to the underlying evidence base to instill confidence in the credibility of the recommendations. Concerns about the clinical safety of prescribing recommendations were also raised; it was suggested that safety assurances should be embedded in the EHR.

While many of the comments in workshop 3 echoed the participants of workshops 1 and 2, particularly around the importance of good usability and linking back to the evidence base for recommendations, several safety concerns were also raised. Specific risks and concerns focused on protecting prescribers from legal challenges if recommendations were not correctly followed. To mitigate these risks, participants advised enabling a full audit trail of past decisions to be reviewed and suggested that it should be possible to record the reasons for overriding prescribing recommendations.

**Table 2:** Summary of key themes identified across 3 user workshops.

Workshop number	Theme	Summary
1	Usability	Pharmacogenetics must be easy to access and integrated into the EHR^a^. There are multiple EHRs
1	Usability	Messaging around pharmacogenetics must be clear and accessible; directions must be easy to follow even by trainees
1	Integration into clinical pathway	Multiple options should be available–flags in EHR and a thorough review of a patient list
1	Implementation complexity	Time is critical for prescribers; they are very busy
1	Incentivization	Incentivization for pharmacogenetics is important
1	Implementation complexity	Do not underestimate how complex this will be to implement in the real world
1	Importance of pharmacogenetics	There are compelling arguments why people would want to adopt this
2	Integration into clinical pathway	Pop-ups and review lists with pharmacogenetics information are useful
2	Incentivization	Incentivization for pharmacogenetics is important; ideally, incentivization would come from a national level
2	Integration into clinical pathway	Integrate with existing systems and mechanisms where possible. Where exactly to locate within the EHR was not universally agreed and noted that prescribers interact differently with the EHRs
2	Credibility of system	Ensuring trustworthiness and credibility of pharmacogenetics information is important
2	Safety concerns	Reassurances about patient safety are important for people engaging with pharmacogenetics
2	Implementation complexity	Sharing data between EHRs (interoperability) is very difficult–a data infrastructure that supports the movement of pharmacogenetic data between organizations is required
3	Integration into clinical pathway	Confirmation that (1) pop-ups are useful but (2) pop-up fatigue is a problem
3	Implementation complexity	Quality of EHR data can be poor; reliance on EHR data for clinical decision-making can be difficult
3	Credibility of system	Having more detailed information available with links to evidence is very important
3	Risk or safety concerns	Making details of previous pharmacogenetics decisions available is important so that this can be reviewed when prescribing
3	Risk or safety concerns	Overriding pharmacogenetics recommendations should require action that is recorded with clear reasons for overriding
3	Risk or safety concerns	What are the legal consequences of overriding a pharmacogenetics recommendation? How do we protect prescribers?

^a^EHR: electronic health records.

### Iterative Prototyping Outputs

**Figure 3:**
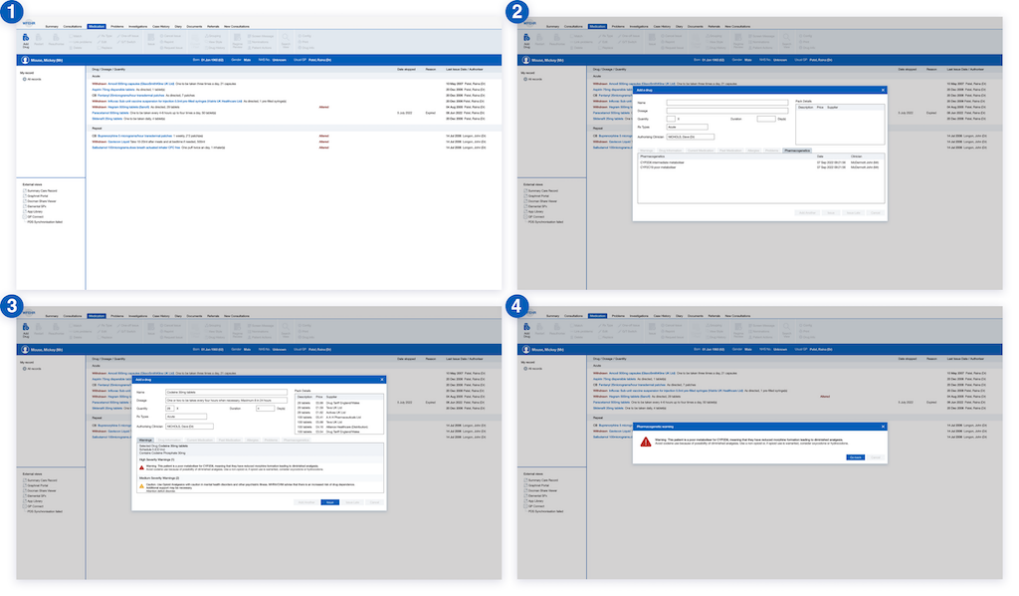
Prototype screens showing where pharmacogenetic results could be surfaced to users. Screen (2) shows the results within the prescribing box and (3) shows specific prescribing guidance surfaced as a “high severity warning” once a medicine has been selected. Screen (4) is an alternative interruptive alert surfacing the guidance if a user ignores or misses the first warning (all data is fictitious).

Across all workshops, participants reviewed high-fidelity design prototypes of how pharmacogenetic recommendations could be integrated into an EHR system. The prototype review of a realistic EHR enabled participants to try out a real-life prescribing scenario and to visually identify any barriers to the designed pharmacogenetics-prescribing process. A screenshot of the prototype is shown in Figure 3. The prototype was iterated and updated between each workshop based on feedback and gathered requirements. We have subsequently publicly published the prototype screens on figshare (Figshare LLC) [20]. Prototyping has subsequently informed the business case for the procurement of a technology partner to develop the technical solution to implement this system as part of the ongoing PROGRESS project.

## Discussion

### Principal Findings

This study applied a co-design approach to better understand the needs and requirements of frontline clinicians for a pharmacogenetic clinical service. We identified that, in order to be used in practice, pharmacogenetic data must be incorporated within existing EHRs and prescribing workflows. The information presented to primary care clinicians must be clear and understandable as the service will be used by a range of end users with varying knowledge of pharmacogenetics. Finally, the prescribing recommendations should be authoritative, with direct links to the underlying evidence base to provide confidence in the validity of results. Based on these functional requirements, we designed an interactive prototype, which demonstrates pharmacogenetic clinical decision support integrated with a primary care EHR system. This study demonstrated a novel method to overcome the barriers to the implementation of pharmacogenetics in routine clinical practice.

### Prescribing Clinical Decision Support Systems in Primary Care

Clinical decision support systems play a significant role in primary care with most EHRs providing a range of alerts to help clinicians make better, evidence-based decisions. They have been shown to improve the quality and safety of care with a recent review by Sutton et al [21] suggesting a potential reduction in patient harm. However, the authors also caution against the challenges of designing and developing systems that are adopted and have a sustained impact [21].

Several prescribing clinical decision support systems have been reported in primary care. For example, Rogero-Blanco et al [22] describes the implementation of a tool to identify patients at risk of adverse drug reactions due to polypharmacy. This tool was embedded within a single EHR and worked on an overall patient list, rather than individual patient records [22]. An example of active alerts was implemented by van Staa et al [23] to inform antibiotic prescribing in primary care with a view to improve adherence to best practice guidelines. Although such tools are useful, their impact on care and outcomes can be limited. A recent meta-analysis on clinical decision support systems by Kwan et al [24] showed only small to moderate improvements in clinical endpoints. In addition, due to the significant heterogeneity, it remains challenging to define the predictors of impactful clinical decision support systems [24]. NHS England recently published national guidance for the implementation of clinical decision support systems in the NHS, highlighting a design-led approach as 1 of the key recommendations for success [25].

This study is the first of its kind to apply an iterative codesign methodology to inform the needs and requirements of a pharmacogenetic clinical decision support system. This method has been shown to work in other clinical areas and by working with end users, we generated ideas that may reduce barriers to adoption [26]. By converting these into high-fidelity prototypes, we were able to drive visual engagement with workshop participants resulting in valuable feedback. This has established the user requirements of a pharmacogenetic clinical decision support system and also helps to inform the technical back-end infrastructure and integrations required to realize the desired user experience.

### Implications for Clinical Practice

The evidence for the impact of pharmacogenetic-based prescribing is well-established and continues to expand. The recent Pre-emptive Pharmacogenomic Testing for Preventing Adverse Drug Reactions (PREPARE) study found, as part of an international multicenter randomized controlled trial in nearly 7000 participants, that pharmacogenetics reduced the risk of adverse drug events by at least 30% [27]. The authors make a compelling case for widespread adoption and development of infrastructure that supports clinical implementation. In the United Kingdom, the joint Royal College of Physicians and British Pharmaceutical Society report on pharmacogenetics calls for personalized testing for the safety and effectiveness of common medicines to be offered throughout the NHS [28]. Our study provides a critical perspective from frontline clinicians who would be expected to use pharmacogenetics as it becomes more mainstream and part of routine clinical practice. This aligns with the literature that highlights the clear requirement to integrate pharmacogenetic data within EHRs to drive clinical decision support systems [29]. We identified that interoperability and data sharing across health care organizations remains a key implementation challenge for personalized medicine.

For health IT solutions to effectively impact patient care, they must provide a view of clinical data that complements the clinical workflow [30]. It became clear through prototyping that primary care clinicians did not consider being able to view pharmacogenetic results on a routine basis as a priority. Pharmacogenetics only became relevant during the prescribing workflow. Furthermore, to provide meaningful clinical decision support, interruptive alerts were only relevant if the prescriber selected a drug for which the patient had a significant gene-medicine interaction, and a therapeutic implication was relevant. This complements previous work by Keeling et al [31], that merely storing pharmacogenetic data as static information in the EHR does not maximize the benefits of the intervention. Rather, querying a patient’s pharmacogenetic data and returning relevant results at the time of prescribing enhances the potential for greater quality decision-making and complements the current workflow.

The findings from this study suggest that clinicians are eager to engage and use pharmacogenetic results to inform prescribing decisions. The general awareness of the roles genomics plays in the delivery of routine clinical services is increasing. A systematic review by Qureshi et al [32] on the barriers and facilitators of pharmacogenetics in primary care suggested that implementation may be facilitated by educating clinicians about the benefits of pharmacogenetic testing. A UK-based study on perceptions of primary care clinicians revealed interesting concerns about the cost-effectiveness of pharmacogenetics and the risks of direct-to-consumer consumer testing [33]. Reflecting on these findings as well as on the results from a structured review of implementation models by Hayward et al [10], it is clear that any pharmacogenetic program in primary care must have an educational component as one of its core pillars in order to be successful [10].

### Implications for Policy and Strategy

Based on the functional needs to provide a query-response of pharmacogenetic data in a disparate landscape of EHRs, a decoupled architecture, which separates data from application may provide the appropriate infrastructure to implement pharmacogenetics at scale. A vendor-neutral data platform for pharmacogenetic data that uses standards-based application programming interfaces may allow a range of EHR and clinical decision support system providers to integrate pharmacogenetics within workflows. As highlighted by Blagec et al [34] as part of the Ubiquitous Pharmacogenomics consortium, this is dependent on the development of robust data standards that allow pharmacogenetic data to be stored in an open and structured manner [34]. Building on this, a systematic review on the inclusion of standards in the implementation of pharmacogenetics by Roosan et al [35] found that only 9 out of 32 articles mentioned standards, with only 4 providing solutions for the lack of interoperability. The authors discussed how interoperability is clearly essential for widespread implementation of pharmacogenetics, and how a lack of focus on standardized data remains a significant challenge [35]. Policymakers should thus promote the development standards including open EHR data models and Fast Healthcare Interoperability Resources through established standards development organizations such as the Global Alliance for Genomics and Health (GA4GH) [36].

The usability of EHRs is known to impact clinicians’ experience of practice and the potential risk of burnout [37]. It is therefore imperative to design solutions that exert minimal additional cognitive load on end users while maximizing the added value technology can provide to clinical workflows. By involving clinicians early in our study, we identified potential barriers, which may be overcome by adopting a user-centered approach to the design process. This included presenting pharmacogenetic data within existing workflows and supporting clinical decision-making in real-time. Such so-called “active” clinical decision support systems have been reported extensively in the literature, including their application in pharmacogenetics [38]. However, implementers must be conscious of the risk of alert fatigue. Clinicians presented with inappropriate alerts, or an overwhelming number of alerts may distrust, disagree with, or ignore them [39]. Alerts should appear at the right time and provide context-relevant information that enhances clinical decision making, in a practical, standardized format. Importantly, prescriptive recommendations need to be reflective of local formulary and agreed upon by appropriate medicines optimization committees and medicines governance bodies to limit the burden of conflicting alerts. Future pharmacogenetic clinical decision support systems should publish data on alert responses (acceptance vs override) to provide a real-world evaluation of the effectiveness of the implementation.

### Limitations

We sought input from health care professionals across the United Kingdom during this study. However, having included a limited number of participants, the generalizability of our findings may be limited. Results will particularly apply to a UK public health care setting, with a focus on primary care, rather than secondary care, where requirements may slightly differ. Alternative methods, which capture more responses across a wider range of users and geographies, such as surveys, may be used in the future to complement and validate this study. We used remote think-aloud methods for our ethnographic observations through screen sharing, and, as a result, we may not have been able to fully capture additional details, such as the physical environment or distractions within the clinic room. Finally, the areas of genomics and digital health are fast evolving [40]. The way prescribing clinical decision support may be implemented may change with emerging technology or service models, such as patient portals or tools embedded within pharmacies rather than at the point of prescribing.

### Conclusion

The implementation of pharmacogenetics in primary care has the potential to improve patient care and save health care costs. This study demonstrated how systems could be designed that are acceptable to primary care clinicians increasing the probability of achieving intended benefits. Active clinical decision support systems (alerts) are likely to personalize medication selection for patients if implemented within existing EHRs and designed to present information in a practical and standardized format. To achieve this, a decoupled architecture, which separates data storage from clinical application is preferable as it will allow the integration of pharmacogenetics across a range of EHRs using application programming interfaces. Alongside user experience and technical development, there remains a need to synchronously educate and upskill nongenomics health care professionals in pharmacogenetics and integrate within routine medicines governance pathways to achieve widespread adoption and impact on care outcomes.

## Appendix

Multimedia Appendix 1 National Health Service Research Ethics Committee decision tool result.

## Abbreviations

EHR: electronic health record
GA4GH: Global Alliance for Genomics and Health
GP: general practitioner
NHS: National Health Service
PREPARE: Pre-emptive Pharmacogenomic Testing for Preventing Adverse Drug Reactions
PROGRESS: Pharmacogenetics Roll Out: Gauging Response to Service
